# Editorial: Women in psychiatry 2022: computational psychiatry

**DOI:** 10.3389/fpsyt.2023.1167498

**Published:** 2023-04-24

**Authors:** Laura K. M. Han, Danai Dima, Xi Zhu, Lianne Schmaal

**Affiliations:** ^1^Centre for Youth Mental Health, The University of Melbourne, Parkville, VIC, Australia; ^2^Orygen, Parkville, VIC, Australia; ^3^Department of Neuroimaging, Institute of Psychiatry, Psychology and Neuroscience, King's College London, London, United Kingdom; ^4^Department of Psychology, School of Health and Psychological Sciences, University of London, London, United Kingdom; ^5^Department of Psychiatry, Columbia University Medical Center, New York, NY, United States; ^6^Department of Psychiatry, New York State Psychiatric Institute, New York, NY, United States

**Keywords:** women in computational psychiatry, brain age, decision-making, Amazon Mechanical Turk (MTurk), psychiatry

Despite the growing representation of women in research, their involvement in scientific journal content is consistently too few (1). Gender disparities are visible in the unequal distribution of advanced doctoral degrees, research positions, and success rates of major grants, leading to persistent underrepresentation of women in the medical field (2, 3). To achieve equity, diversity, and inclusion in computational psychiatry, continued efforts to support the advancement of women are needed. As part of such efforts, the Frontiers Research Topic series, “*Women in Psychiatry: Computational Psychiatry*” was launched. This editorial will provide a brief overview of the involvement of women in the five studies featured in this series, before putting their research findings into context.

Five peer-reviewed research articles from authors affiliated with Asian, Oceanian, European, and North American universities were included in the current Research Topic. Of these contributions, 60% were handled by female editors. An equal number of female and male reviewers participated in the peer-review process. In terms of authorship, 64% of all authors, 70% of corresponding authors, and 80% of lead authors were women. This higher representation of women promotes and increases the visibility of female colleagues, and we are proud to feature five diverse contributions to the field, all first and/or last-authored by women. Studies were performed across a wide variety of populations, ranging from healthy individuals to psychiatric populations with obsessive compulsive disorder (OCD), attention deficit/hyperactivity disorder (ADHD), and alcohol use disorder (AUD).

Several innovative computational methods were deployed to investigate biological and cognitive processes in psychiatric disorders. First, Kurth et al. examined normative age-related structural brain patterns and found preliminary evidence for “younger appearing” brains in a sample of children with ADHD, compared to healthy controls, potentially providing an explanation of the characterization of ADHD as a neurodevelopmental disorder. Future research is needed to replicate the findings and to conduct more comprehensive studies linking multiple aspects of ADHD. Second, Baǧci et al. used a probabilistic reversal learning task to understand the mechanisms behind impaired decision-making in AUD. The researchers found that patients showed general impairments in learning and decision-making, but no evidence of increased perseveration rates after reversals. Patients showed enhanced learning from negative feedback, a tendency toward reduced learning from positive feedback, and more random behavior than healthy controls. These findings emphasize the benefits of reinforcement learning models in understanding impaired decision-making in AUD patients. Third, Chen et al. used a probabilistic reasoning task to study and compare decision-making in OCD patients and healthy controls. They found that OCD patients took longer to respond than healthy controls, although similar overall accuracy was achieved. The study sheds light on the decision-making process in OCD patients and suggests that the deficiency in their decision-making is due to inefficient accumulation of evidence, rather than a sensory perception deficit. Targeting the cortical-basal-ganglia circuitry involved in decision-making may improve their ability to make confident and accurate decisions. Additionally, the study highlights the importance of using decision-making tasks to better understand the underlying neural mechanisms of decision-making in both healthy individuals and clinical populations. Together, these articles showcase how we can use computational approaches to advance our understanding of the brain and behavior in psychiatric illness.

Two studies utilized Amazon's Mechanical Turk (AMT) to study COVID-19 related factors, highlighting the trend for online psychiatric research. Mills-Finnerty et al. used Bayesian computational modeling to examine cognitive performance in AMT workers pre- and peri-COVID. The authors reported no significant increase in mental health symptoms (depression and anxiety) during the pandemic compared to pre-COVID, however, younger AMT workers reported higher symptom severity and worse cognitive performance. Older adults showed relatively preserved cognitive performance and a mildly beneficial effect of subclinical anxiety and depression symptoms. Increased access to mental health services, particularly telehealth or smartphone-based service could help mitigate negative impacts on their wellbeing and long-term health outcomes. Todorovic et al. used machine learning to identify (COVID-related) predictors of child maltreatment and intimate partner violence during the pandemic. The study found that parents' psychological distress and lack of children's outdoor activities were significant contributors to child maltreatment, whereas factors related to strict social distancing or lockdown were less important. Empathic concern, emotion regulation, and non-conflictual relationships between parents were identified as critical objectives in efforts to prevent child maltreatment. While AMT provides scientists with a revolutionary way to crowdsource research data, a recent study confirmed that the prevalence of, for example, major depressive disorder, generalized anxiety disorder, and AUD in online community of AMT workers is twice as high compared to the general population (4). The development of guidelines and data quality measures will be crucial to limit systematic biases in research. Despite these challenges, big data are important for computational psychiatry, and AMT is a valuable tool that could be used to generate promising findings that should be further studied in more controlled studies.

The work presented here highlights the diversity of research performed across the entire breadth of Psychiatry research and presents advances in theory, experiment, and methodology with applications to compelling societal issues. We hope it inspires you to create, cultivate, and celebrate a research culture that is healthy for science and all scientists.

## Author contributions

LH: writing—original draft. DD, XZ, and LS: writing—review and editing. All authors contributed to the article and approved the submitted version.
